# Satellite Glial Cells in Human Disease

**DOI:** 10.3390/cells13070566

**Published:** 2024-03-23

**Authors:** Menachem Hanani

**Affiliations:** 1 Laboratory of Experimental Surgery, Hadassah-Hebrew University Medical Center, Mount Scopus, Jerusalem 91240, Israel; hananim@cc.huji.ac.il; Tel.: +972-2-5844721; 2 Faculty of Medicine, Hebrew University of Jerusalem, Jerusalem 91120, Israel

**Keywords:** dorsal root ganglion, trigeminal ganglion, satellite glial cells, sensory neuron, autoimmune diseases, herpes, fibromyalgia, rheumatoid arthritis

## Abstract

Satellite glial cells (SGCs) are the main type of glial cells in sensory ganglia. Animal studies have shown that these cells play essential roles in both normal and disease states. In a large number of pain models, SGCs were activated and contributed to the pain behavior. Much less is known about SGCs in humans, but there is emerging recognition that SGCs in humans are altered in a variety of clinical states. The available data show that human SGCs share some essential features with SGCs in rodents, but many differences do exist. SGCs in DRG from patients suffering from common painful diseases, such as rheumatoid arthritis and fibromyalgia, may contribute to the pain phenotype. It was found that immunoglobulins G (IgG) from fibromyalgia patients can induce pain-like behavior in mice. Moreover, these IgGs bind preferentially to SGCs and activate them, which can sensitize the sensory neurons, causing nociception. In other human diseases, the evidence is not as direct as in fibromyalgia, but it has been found that an antibody from a patient with rheumatoid arthritis binds to mouse SGCs, which leads to the release of pronociceptive factors from them. Herpes zoster is another painful disease, and it appears that the zoster virus resides in SGCs, which acquire an abnormal morphology and may participate in the infection and pain generation. More work needs to be undertaken on SGCs in humans, and this review points to several promising avenues for better understanding disease mechanisms and developing effective pain therapies.

## 1. Introduction

### 1.1. Satellite Glial Cells in Sensory Ganglia

**Figure 1:**
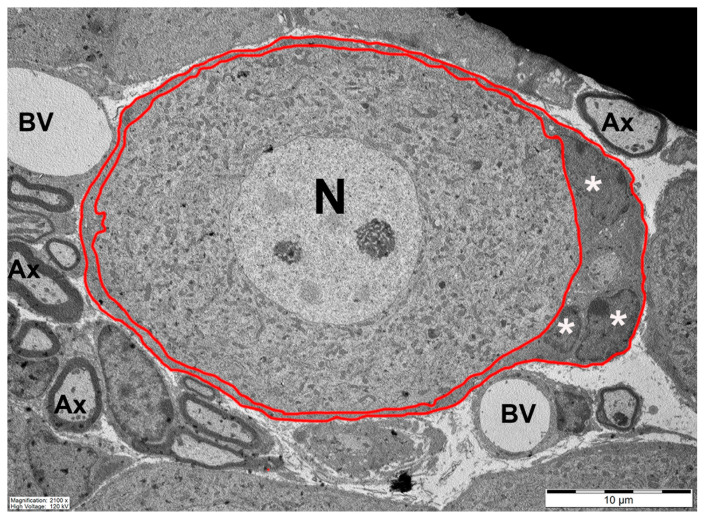
The neuron–SGC unit. A low power electron micrograph showing SGCs (marked red) surrounding a neuronal soma (N) in mouse trigeminal ganglion. Three nuclei of SGCs are marked with asterisks (*). BV, blood vessel; Ax, axon.

Sensory ganglia contain the cell bodies of sensory neurons, which send one process to the periphery and another to the central nervous system (CNS). The main sensory ganglia are dorsal root ganglia (DRG), which innervate most parts of the body, including many internal organs; trigeminal ganglia (TG), which innervate the face, teeth, and part of the scalp; and nodose ganglia, which innervate many internal organs (lungs, heart, most of the gastrointestinal tract, and others). Abnormal electrical activity in the neurons of sensory ganglia is a major factor in chronic pain [1]. The main type of glial cells in sensory ganglia are satellite glial cells (SGCs), which surround individual neurons completely, forming a functional neuron–SGC unit (Figure 1). The organization of SGCs around neurons in human sensory ganglia is similar to that shown in Figure 1. However, as human sensory neurons are much larger than those of mice, the number of SGCs is accordingly greater. This correlates with studies showing that the number of SGCs surrounding a neuron increases with the size of the neuron [2]. Images of neurons and SGCs are displayed in several figures of human ganglia shown below. In some cases, small groups of neurons are surrounded by a common SGC sheath [2,3,4]. The distance between the SGC and neuron is about 20 nm, which enables effective chemical communication between the two cell types; for reviews on SGCs, see [3,4,5,6,7,8,9,10,11]. SGCs are also present in sympathetic and parasympathetic ganglia, but these are not covered in the present review.

Sensory ganglia have been investigated for many years with the aim of learning about their normal function as the first station of the somatosensory and trigeminal pathways and their role in acute and chronic pain. Chronic pain is a major global public health problem, which afflicts up to 19% of the adult population [12], and there is no effective treatment for many of the patients. There is firm evidence that peripheral injury or inflammation induces pathological changes in the axons, terminals, and somata of sensory neurons. Such changes may augment the excitability of the neurons and cause excessive firing of action potentials, which will be sensed as nociceptive signals at the spinal cord [1,13,14]. There is evidence from clinical studies in humans that, in several types of chronic pain states (e.g., postherpetic pain, trigeminal neuralgia, phantom limb pain), the main underlying mechanism is abnormal firing in sensory neurons [1,15,16]. Therefore, more attention should be directed to the periphery when considering chronic pain.

Glial cells are essential for the normal development and function of the nervous system. They have a protective role, but they may also contribute to pathological states [17,18,19]. For example, prolonged astrocyte reactivity during autoimmune or neurodegenerative disorders can lead to excessive inflammation [18]. Work on the CNS has indicated that glial cells play important roles in a large number of neurological diseases, including Alzheimer’s disease, stroke, and Parkinson’s disease [17,20,21]. In addition, there is considerable evidence that microglia and astrocytes in the CNS contribute to pain induced by peripheral injury [22,23,24]. The most relevant change in glial cells under pathological conditions is their activation, or gliosis [18,25]. Activated glia release molecules such as proinflammatory cytokines, which can increase neuronal excitability, which may have a role in nociception.

In contrast to the extensive research on glial cells in CNS, there has been little interest in SGCs, and only the last two decades have seen progress in the study of these cells. It is now established that the activation of SGCs can lead to the sensitization of sensory neurons, and that SGCs must be taken into account when considering pain mechanisms. Most of the knowledge on SGCs has been derived from animal studies, and information on the role of SGCs in human disease is scarce. The main objective of this review is to highlight the current information on SGCs in human sensory ganglia and their possible roles in disease. The account below focuses on DRG and TG, and nodose ganglia are not mentioned, as current knowledge on SGCs in these ganglia in humans is minimal. This topic could be of considerable interest, as the nodose ganglia give rise to the vagus nerve, which controls numerous autonomic functions and mediates essential reflexes, and whose stimulation is used for treating various disorders. There is evidence that SGCs in mouse nodose ganglia are altered profoundly in a mouse model of systemic inflammation [26], and this may happen in human disease as well.

Several fairly specific molecular markers for SGCs have been identified, and the main ones are glutamine synthetase, the K^+^ channel Kir4.1, small conductance Ca^2+^ activated K^+^ channels (SK3), glutamate-aspartate transporter (GLAST1, or EAAT1), connexin 43, and fatty acid binding protein 7 (Fabp 7) [9]. Glial fibrillary acidic protein (GFAP) is upregulated in SGCs under pathological conditions and can serve as a marker for SGC activation.

### 1.2. SGCs and Chronic Pain

#### Sensory Ganglia in Humans

For many decades, research on sensory ganglia focused on rodents. This has led to major progress in the understanding of the physiology and pharmacology of sensory neurons [14,27]. Recently, with the establishment of human tissues banks, work on human ganglia became possible, which yielded several studies on these tissues. The available reports demonstrated some differences between neurons in human and rodent DRG. For example, Rostock et al. [28] found both similarities and differences between human and mouse neurons: peptidergic nociceptors (expressing the high affinity nerve growth factor receptor *TrkA*) expressed *trpv1* at a greater extent in human than mouse neurons. Also, co-expression of the Na^+^ channels Na_v_1.8 as well as Na_v_1.9 in TRKA neurons was greater in human than mouse DRG.

Körner and Lampert [29] compared a large number of electrophysiological studies on rodent DRG with the three available ones on humans. They reached the conclusion that not all the categorization schemes used in rodents are applicable to human DRG. For example, isolectin B4 (IB4) labelling has been used extensively to distinguish subgroups of rodent DRG, but human primary sensory neurons do not bind IB4. Another difference is that, whereas in rat DRG small neurons are classified as nociceptive (based on the presence of tetrodotoxin-resistant Na^+^ channels), this does not hold for human DRG. Avraham et al. [30] carried out a direct comparison of the transcriptional profile of SGCs in mice, rats, and humans at the single-cell level. They found similarities between key features in rodents and humans, such as enrichment for lipid metabolism and peroxisome proliferator-activated receptor alpha (PPARa) signaling. Still, they noted differences in ion channels and receptor expression, which may imply differences in SGC–neuron communication and functions in pain conditions.

Acid-sensing ion channels (ASICs) play a critical role in nociception. It has been found that the distribution of ASIC 1, 2, and 3 in human DRG was quite different from that in rodents [31]. For example, in mice, the expression pattern depended on neuronal size and type, whereas this was not observed in human DRG. The authors commented that the expression differences between humans and rodents should be taken into consideration when evaluating the translational potential of rodent studies.

Only little is known about the pharmacology of neurons and SGCs in humans. As calcitonin gene-related peptide (CGRP) is highly relevant to migraine, Eftekhari et al. [32] examined CGRP and its receptors in TG of rats and humans. They found that neurons in both rats and humans contained CGRP and its receptors, whereas SGCs in both species expressed only the receptors. This suggests that CGRP may serve for neuron-to-SGC signaling in TG and play a role in migraine. Anti-CGRP monoclonal antibodies (CGRP-mAbs) are being used for migraine treatment. Noseda at al. [33] injected fluorescently labeled CGRP-mAB into rats to locate its site of action and showed that it did not cross the blood–brain barrier but was present in sensory and autonomic ganglia. As both sensory neurons and SGCs express CGRP receptors, it is likely that the antibody acts on ganglionic cells, including SGCs.

Jia et al. [34] compared the transcriptome in TG of mice and humans, and found that the gene expression of purinergic receptors, connexins, pannexins, and neurotransmitter transporters was qualitatively similar, but there were some quantitative differences between the two species. Using immunohistochemistry, Koeppen et al. [35] showed that SGCs in human DRG share several functionally important proteins with rodents: connexin 43, glutamine synthetase, metabolic glutamate receptors type2/3, EAAT1, GFAP, and the K^+^ channel Kir4.1, which is specific for SGCs and has a major functional role. They also identified the structural proteins, vimentin, S100, and laminin within SGCs; see also Section 2.5.1. Chessell et al. [36] reported that purinergic P2X7 receptors, which are located predominantly in SGCs, play a role in neuropathic pain in both humans and mice. For a comprehensive review of human DRG, see Haberberger et al. [37].

**Figure 2:**
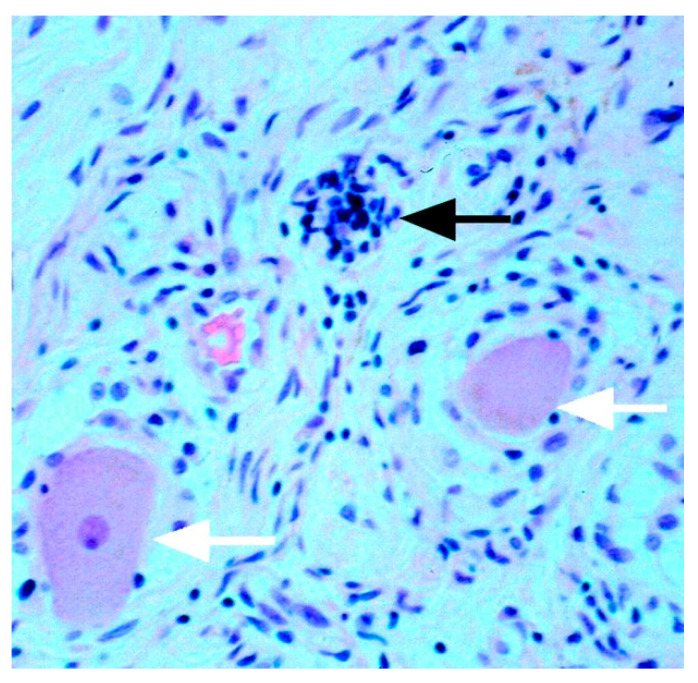
Nodule of Nageotte in human DRG (patient with ganglionitis). The black arrow points at a cluster of SGCs that constitute a nodule. The white arrows indicate normal neurons, which are surrounded by SGCs (their nuclei are stained blue). Reproduced with permission from Rees, J. Neurol. Neurosurg. Psychiatry [41], published by BMJ, 2004.

A hallmark of neuronal damage and death in human sensory ganglia is the formation of Nageotte nodules, which are clusters of SGCs that occupy the space of degenerated neurons (Figure 2). Scaravilli et al. [38] described Nageotte nodules in the DRG of humans of all ages, particularly in aging ones, but did not observe the nodules in DRG from rats of any age or from rats following sciatic nerve axotomy. In contrast, there are reports on the presence of Nageotte nodules in rats that had been injected with the anti-cancer drug paclitaxel [39] and in rats that were poisoned with mercury [40]. This discrepancy may be due to the different types of insults used in the animal studies, but this topic needs to be revisited. It was claimed that Nageotte nodules contain activated SGCs and macrophages, but the functions of these cells have not been investigated. If indeed SGCs in Nageotte nodules are activated, they might release proinflammatory molecules that may alter the function of neighboring SGCs and neurons. This question is worthy of a thorough investigation.

Taken together, these studies indicate that many molecular features are shared between sensory ganglia in humans and rodents. There are no clear-cut conclusions on the validity to humans of pain research in rodents, but the limited available information is encouraging, as it indicates clear parallels between the ganglia in humans and rodents, which vindicates the use of rodents in pain research. Still, work on human tissue should be pursued as much as possible (within the ethical limitations). Some of the studies described below combined experiments on human and rodent material; for example, injecting serum from human patients suffering from pain syndromes into mice, or incubating mouse DRG with such serum, can provide valuable insights into disease mechanisms and therapy. An alternative to rodent models is the use of human induced-pluripotent stem cells (iPSCs) in culture [42]. This method has already yielded interesting results about the properties of mutant Na^+^ channels related to a pain syndrome [43]. In any case, when discussing animal pain models, we need to keep in mind that, even though the biology of humans and rodents is somewhat similar, pain in humans is influenced by cognitive, psychological, and cultural factors, which may limit the translational value of animal work.

## 2. SGCs in Specific Human Disorders

There is unequivocal evidence that, in laboratory animals, SGCs are altered in a large number of pathological states and that these cells are likely to be involved in chronic pain states. In contrast, little is known on changes in SGCs under pathological conditions in humans. In the last few years there has been some progress on this topic, which is detailed below.

### 2.1. SGCs in Viral Diseases

Humans may be afflicted with numerous viral diseases, many of which are associated with pain that can be severe. The following is a survey of the possible roles of SGCs in pain in some common viral diseases.

#### 2.1.1. Herpes

Alpha-herpesviruses, varicella zoster virus (VZV), and herpes simplex virus types 1 and 2 (HSV-1,2), infect the axons of sensory ganglia. After the primary infection (chickenpox in the case of VZV), the virus can reside in a latent form within the ganglia for the life of the host, but can be reactivated, thereby causing disease episodes. Reactivation of VZV results in herpes zoster (shingles), which is characterized by painful skin eruptions that are often followed by post-herpetic neuralgia—a chronic and extremely painful condition [44]. Reactivation of HSV1,2 causes mucocutaneous lesions (cold sores by HSV-1 and genital herpes by HSV-2) and may also result in neuralgia [45]. The role of SGCs in herpetic infection and pain has been controversial. Major questions are whether SGCs are infected during the acute phase and whether they contain the virus during latency. This topic is of clinical relevance because SGCs might act as a barrier for the spread of viruses in the ganglia, and, if they are infected, they may be a site of virus proliferation, which can promote neuronal infection. It has been stated frequently that, during latency, the virus is exclusively located in sensory neurons [46], but there is considerable evidence that it can be found also in SGCs. Croen et al. [47] located VZV in SGCs during latency but not HSV, and Esiri and Tomlinson [48] identified VZV in both neurons and SGCs in a TG from a patient with zoster.

Lungu et al. [49] used in-situ hybridization to examine post-mortem tissues from patients with zoster and found that DRG that innervate sites of reactivation on the skin or adjacent sites displayed strongly positive signals in neurons and SGCs. In contrast, cells in DRG innervating distant sites were rarely positive. Also, VZV DNA was found in both the nuclei and the cytoplasm of neurons innervating areas of zoster, whereas, in neurons innervating zoster-free areas, the DNA was found only in the nuclei of neurons and their surrounding SGCs. This may indicate that the mere presence of the virus in DRG cells is not sufficient for causing zoster. 

**Figure 3:**
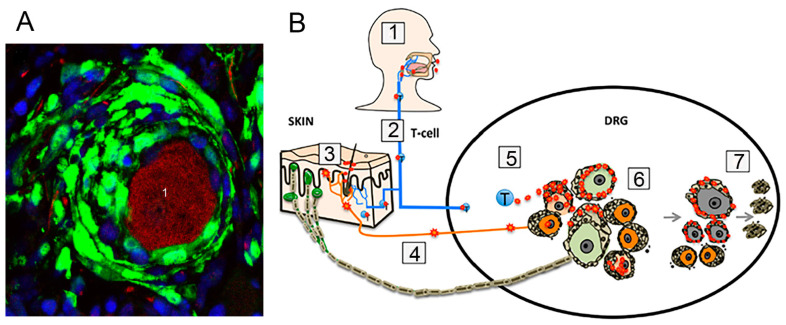
SGCs in VZV infection. (**A**) Micrograph of a neuron (1) labeled red for RT97 (mechanoreceptor marker) surrounded by abnormal onion-like layers of SGCs (labeled green for the VZV protein IE63). (**B**) Diagram depicting two possible modes by which sensory neurons can be infected by VZV. (1) Primary VZV infection is initiated in respiratory epithelial cells, followed by transfer to T cells in lymphoid tissue. The virus is represented as red dots. (2) Infected T cells enter the circulation. (3) T cells carry the virus to the skin. (4) From the skin, T cells reach DRG neurons by retrograde axonal transport. (5) The T cells also reach the DRG directly. Both pathways promote infection of DRG cells. (6) VZV gains access to both nociceptive (small, orange) and mechanoreceptive (large, green) neuronal somata by either route. Replication occurs only in nociceptive neurons. If replication in neurons is uncontrolled, infection of SGC facilitates spread to neighboring neuron–SGC units. (7) Over time, VZV infection in SGC contributes to neuronal death, mostly of mechanoreceptors, which presumably are more dependent on SGCs support than nociceptors. This leads to the formation of nodules of Nageotte. Reprinted from Zerboni and Arvin [50] under Creative Commons Attribution (CC-BY) license from PLOS Pathog.

A major problem in VZV research is that laboratory animals are not infected by it. To overcome the lack of a rodent model of VZV, Zerboni and Arvin [50] grafted pieces of human DRG under the kidney capsule of severe combined immunodeficiency (SCID) mice. They employed this model to find out whether SGCs are infected in the *acute phase*. The human DRG tissues differentiated normally and survived within the mice. The grafted tissues were infected with VZV and were removed 3–20 weeks afterwards and examined histologically. The authors reached the following conclusions:

1. When VZV infects the skin, axon terminals of DRG neurons take the virus up, and it reaches the cell bodies by axonal transport. In patients with compromised immune response (usually older persons), the virus proliferates in the neurons and may cross the narrow gap between neurons and SGCs and infect the SGCs. From SGCs, the virus can propagate to non-infected neurons and infect them as well; thus, the extent of painful skin regions can increase. 2. When the airways are exposed to VZV, T-cells can be infected and may enter the ganglia via the circulation. As SGCs form the first line of neuronal protection, they will be infected, and from them the virus can spread into the neurons (Figure 3). 3. Over time, some infected neurons die, and SGCs phagocytose them. 4. VZV infection disturbs SGC function, which will impair neuronal survival. Moreover, damaged SGCs may also contribute to post-herpetic pain. This account demonstrates the importance of SGCs in the acute phase and emphasizes the concept of the SGC–neuron unit but still does not provide insight into the role of SGCs in the latent phase. Also, though this description may be valid, it ignores the possibility that the virus can cross the permeable SGC cover and reach the neuronal surface.

Another question is whether and how SGCs contribute to herpetic pain. Devor [51] made a case for the importance of DRG in post-herpetic pain, which is consistent with the idea that neuron–SGC interactions contribute to this disorder. One possibility is that SGCs are activated during herpes infection and release proinflammatory cytokines, thereby leading to neuronal excitation and pain [52,53]. However, currently, there is no definitive evidence that SGCs are activated in herpes patients or in animal models, although Ouwendijk et al. [54] reported that, in monkeys infected with simian varicella virus (a VZV model), SGCs were activated, as assayed by increased expression of ‘activation markers’ CD68 and MHC class II. In HSV-1-infected mice, GFAP protein and RNA in DRG were increased, but the cellular location of GFAP was not reported [53]. As the most accepted activation marker in glial cells is the increased level of GFAP, this issue remains open. Nevertheless, the available information suggests that SGCs may play a major role in herpes infection and pain, and more work must be performed on human tissue to test this idea. The question of the role of SGCs in the latent phase is open and also needs to be addressed.

Another central question in herpes research is the mechanism of reactivation and what prevents it from occurring during many years of latency [55]. Reactivation of VZV may occur spontaneously or following one or more triggering factors such as infection, immunosuppression, trauma, and malignancy, but the molecular triggers of VZV reactivation are not well understood. In this context, SGCs (in addition to local immune cells) in the ganglia may play a role [52]. It is interesting to note that, in an early work, it was found that mixed SGC–neuron cultures of human DRG showed a higher degree of neuronal infection than cultures of purified neurons or SGCs, and only in the mixed cultures was there reactivation [56]. This supports the idea that SGCs play roles in both infection and reactivation.

#### 2.1.2. Human Immunodeficiency Virus (HIV-1)

Peripheral neuropathy and pain are common in patients infected with HIV-1, the virus that causes AIDS [57]. Over half of patients with AIDS develop peripheral neuropathy, which is associated with sensory disorders, including pain. The mechanisms underlying pain in AIDS have received only limited attention, and little is known on the possible involvement of sensory neurons or SGCs in the pain phenotype. In sensory ganglia of AIDS patients, it is common to find Nageotte nodules [58]. Brannagan et al. [59] used PCR to amplify DNA for in-situ hybridization and found that HIV-1 virus was located in SGCs, and occasionally in neurons in DRG from AIDS patients who suffered from neuropathy, but not in AIDS patients without neuropathy. The large nerve fascicles in the DRG sections were always negative for HIV DNA and RNA, even in the presence of many HIV-positive cells in the DRG, which is due to the greater permeability of the blood–nerve blood barrier in the ganglia compared with nerve fibers. These findings do not prove a causative role for SGCs in neuropathy in AIDS but are highly suggestive of such a role.

Support for the involvement of SGCs in HIV-associated pain has been obtained in experiments where the HIV envelope glycoprotein 120 (gp120) combined with ddC (2′,3′-dideoxycytidine) were injected into rats [60]. This treatment activated SGCs in DRG, induced pain-like behavior, and increased the expression of P2Y12 purinergic receptor mRNA and protein in DRG SGCs. DRG neurons in the treated rats showed an augmented response to the P2Y12 receptor agonist 2-MeSADP. shRNA against P2Y12 receptors in SGCs decreased the release of pro-inflammatory cytokines, decreased phosphorylation of p38 MAPK, and reduced hyperalgesia in the gp120+ddC-treated rats. Thus, downregulating the P2Y12 receptor relieved hyperalgesia in gp120+ddC-treated rats. This work provides a possible clue to the contribution of changes in DRG to pain in HIV-1.

#### 2.1.3. SARS-CoV-2 (COVID-19)

The SARS-CoV-2 virus (COVID-19) infects the mucosa of the respiratory tract and may cause fatal disease. The virus binds to cells mainly via angiotensin converting enzyme (ACE-2), which allows the virus to enter the cells and infect them. In addition to respiratory distress, patients display gastrointestinal, renal, hematological, and other symptoms. COVID-19 patients also suffer from a variety of neurological problems, including pain and loss of the senses of smell and taste [61]. These symptoms may afflict patients in both the acute phase and the long-term (long COVID). Headache is one of the most common neurological symptoms in COVID-19 [61,62]. COVID-19 RNA was detected in the TG of COVID-19 patients [63], which may be related to the high prevalence of headache in these patients.

**Figure 4:**
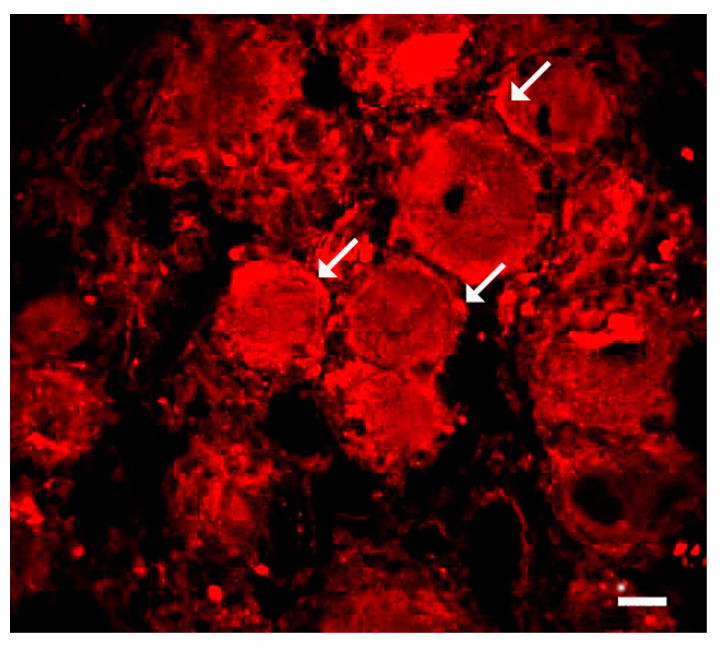
Human DRG immunostained for ACE-2. The arrows point at cells that might be SGCs. Scale bar, 20 µm. Reprinted from [64] under Creative Commons Attribution (CC-BY) license, Pain Rep., LLW, 2021.

It has been reported that, in human DRG, neurons as well as “some” SGCs carry ACE-2 [64]. Work on human TG also showed that neurons were ACE-2-positive [65], but an examination of the images suggested that SGCs were positive as well (Figure 4). Thus, the virus may infect SGCs first and then can pass on to the neurons, as was proposed for VZV [50], as described above. These results are in accord with a study using a model for COVID-19 infection in golden hamsters, where the viral RNA was detected by in-situ hybridization in SGCs of DRG [66].

To account for the olfactory deficits and headache in COVID-19, it has been suggested that, under the influence of the virus, SGCs in the TG release cytokines that may influence the sensory neurons [67], but this needs to be tested. It may be suggested that SGCs play a role in the infection of sensory neurons with COVID-19 virus and thus are involved in the pain syndromes in this disease, but this has to be confirmed experimentally.

### 2.2. SGCs in Autoimmune Diseases

In a comprehensive review, Xu et al. [68] made a strong case for the role of immunoglobulins G (IgGs) in pain syndromes. IgGs can stimulate nociceptive receptors by either binding the constant region (Fc) or antigen-binding (Fab) region at axons (nodes of Ranvier) or neuronal somata. The binding of IgG complexes to Fc-gamma receptors on nociceptive DRG neurons can evoke nociceptive activity without the occurrence of inflammation or injury [69]. Immune cells such as T lymphocytes and macrophages can be present in sensory ganglia, and, under pathological conditions, increase the synthesis and release of proinflammatory cytokines that can sensitize the neurons. The possibility that IgG can target SGCs has received minimal attention. However, as SGCs are the first to be exposed to molecules or pathogens that enter the ganglia, it is conceivable that they might be involved in the nociceptive signaling in the ganglia. Clearly, immune cells in the ganglia, as well as central sensitization, also participate, but SGCs cannot be ignored.

There is evidence that SGCs in humans display some characteristics of immune cells. van Velzen et al. [70] reported that SGCs function as antibody-presenting cells (APC) and can also perform phagocytosis. Like immune cells, SGCs can undergo activation in response to injury and to various neuronal signals, and, while activated, they can release proinflammatory molecules, such as TNFα and IL-1β [3,8]. It is therefore of interest to ask whether SGCs play a role in autoimmune disorders, where antibodies produced by the body can attack some of the body’s cells, thereby inhibiting normal processes or inducing injury. Below, the involvement of SGCs in some such diseases are described briefly.

#### 2.2.1. Rheumatoid Arthritis

Rheumatoid arthritis (RA) is an inflammatory autoimmune disorder that often affects the hands and feet, causing joint pain and stiffness. Other symptoms may be present, and additional organs may also be affected. This is a common disorder with a prevalence of 0.5–1.0% [71,72]. Females are 2–3 times more prone to RA than males [72]. It is believed that the pain in RA is initiated by local inflammation, and that after the inflammation subsides, the main mechanism underlying the pain resides in the CNS in the form of central sensitization. Sunzini et al. [73] stated that “the classic ‘top down’ nociplastic dimension will likely dominate and will continue to be used to explain why ~50% of patients with RA report clinically important levels of pain despite achieving full remission of their systemic inflammatory disease”. (Nociplastic pain is defined as pain that is mechanistically distinct from nociceptive pain, which is caused by ongoing inflammation and damage of tissues, and neuropathic pain, which is caused by nerve damage [74].) This interpretation may be partly correct, but it ignores the possibility that even after the inflammation is resolved, the peripheral nervous system may be still sensitized, and abnormal firing of sensory neurons will persist. Support for this claim was obtained by Wigerblad et al. [75], who tested the actions of autoantibodies against citrullinated proteins (ACPA), which are found in the serum of many RA patients. They found that ACPA induced pain behavior in mice, without causing inflammation. Another example is intestinal inflammatory pain, which can persist even after the inflammation has resolved—a phenomenon called “postinflammatory pain” [76]. This is consistent with work on animal models; we have shown that four weeks after the induction of systemic inflammation in mice, the pain threshold was still low and some changes in SGCs persisted [77]; see also [78].

**Figure 5:**
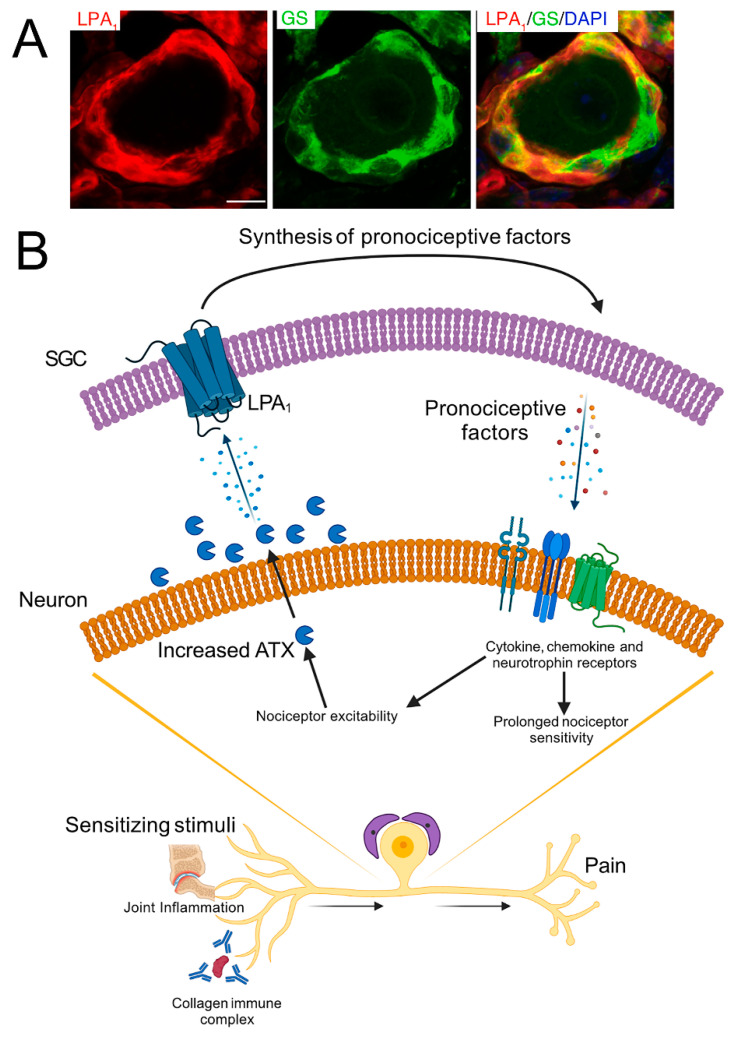
Neuron–SGC interactions in RA. (**A**) Sections of human DRG showing immunostaining for LPA1, the SGC marker glutamine synthetase (GS), and the nuclear dye DAPI (blue). The combined image (right) shows clear colocalization of the LPA receptor LPA1 and GS in SGCs. Scale bar, 50 µm. (**B**) Schematic of lysophosphatidic acid (LPA) signaling in collagen antibody-induced arthritis (CAIA). In the CAIA model, autotaxin (ATX) is elevated in DRG neurons, possibly in response to sensitizing stimuli such as joint inflammation and immune complex activation of nociceptors. ATX increases the levels of LPA, which activates SGCs that express LPA1, leading to SGC activation. Activated SGCs produce increased levels of pronociceptive cytokines and NGF, which can then act on the corresponding receptors in the neurons. This further promotes nociceptor excitability, leading to the development of chronic pain. Reprinted under Creative Commons Attribution (CC-BY) license from Su et al. [80] Brain Behav. Immun., Elsevier, 2022.

Some information on the possible role of peripheral mechanisms in RA was obtained in studies on animal models, where proteins (collagen, albumin) or anti-collagen antibodies were injected into rodents to evoke RA-like inflammation [79]. Progress in understanding the peripheral component in RA took place in recent studies from the group of Prof. Camilla Svensson, which looked for changes in sensory ganglia in RA. Su et al. [80] used the collagen antibody-induced arthritis (CAIA) model of RA, which is based on injecting mice with antibody against collagen type II (followed by lipopolysaccharide [LPS] injection). This induced joint inflammation and pain-like behavior (mechanical, heat, and cold hypersensitivities). Also, GFAP was upregulated in SGCs in DRG from CAIA mice, indicating their activation. Lysophosphatidic acid receptors (LPA1 and LPA3) are G-protein-coupled receptors that bind the lipid signaling molecule lysophosphatidic acid (LPA). Su et al. [80] found that SGCs from mice, and also from a human subject, express LPA1 (Figure 5). They also found that autotaxin, the enzyme that produces LPA, is upregulated in DRG neurons in CAIA mice. Blocking LPA1/3 reduced CAIA-induced mechanical hypersensitivity. Su et al. [80] concluded that CAIA-induced pain-like behavior by LPA signaling is a *peripheral event*, associated with the DRG and involving increased pronociceptive activity of SGCs, which in turn act on sensory neurons. These results are in agreement with the finding that LPA activated SGCs in mice [81]. Thus, SGCs appear as a key element in the CAIA model. It should be pointed out that most of the observations above were made in mice, but the presence of LPA1 in SGCs in human DRG suggests that the conclusions may hold for humans, but this will have to be verified experimentally.

In a subsequent article, Svensson and co-workers studied some molecular mechanisms underlying pain in RA. There is evidence that a major element in RA is the presence of anti-modified protein autoantibodies (AMPAs) against post-translationally modified citrullinated, carbamylated, and acetylated proteins. Using mice, Jurczak et al. [82] investigated the pronociceptive properties of the monoclonal AMPA 1325:01B09 (B09 mAb) derived from plasma cells of an RA patient. The main findings were: 1. B09 mAb induced pain-like behavior in mice without any apparent signs of inflammation. 2. B09 mAb was retained in the DRG, where it altered neurons, macrophages, and SGCs. 3. Fragment crystallizable gamma receptors (FcγRs) in macrophages are required for the development of B09 mAb-induced pain-like behavior. 4. B09 mAb was found to bind to SGCs in vitro, and, in combination with stimuli such as ATP, enhanced transcriptional changes in DRG cells and released pronociceptive factors from SGCs. This apparently occurs by activation of FcγRs in macrophages, leading to the induction of inflammation-related genes in them and resulting in the release of proinflammatory factors and the induction of neuronal sensitization and nociception. It is noteworthy that no changes in gene expression of pro-nociceptive or proinflammatory factors were observed in the joints, in contrast to the prominent increases in mRNA for many factors that were found in the DRG. In summary, although the physiological mechanisms underlying these findings are still not known, it is clear that autoantibodies that target SGCs may be key elements in the generation of pain in RA.

#### 2.2.2. Sjögren’s Syndrome

Sjögren’s syndrome (SSyn) is a chronic autoimmune disease characterized by inflammation of the exocrine glands, especially the salivary and lacrimal, which may cause oral and ocular dryness [83,84]. Other organs can also be affected, e.g., lung, kidney, liver, and joints. SSyn is much more prevalent in females, with a female-to-male ratio of 9:1. Peripheral neuropathy is quite common in SSyn, with a prevalence of 15%, and it is usually associated with pain and abnormal sensations [84]. Only a little work has been performed to clarify the mechanisms of neuropathy in SSyn. As SSyn is an autoimmune disease, it would have been of interest to look for changes in the immune properties of peripheral tissues in patients and in animal models. It was shown that human SGCs display Human Leukocyte Antigen-DR isotype (HLA-DR) [85], which is usually located in APCs such as macrophages and dendritic cells. The main function of HLA-DR is to present foreign peptide antigens to the immune system in order to elicit T-(helper)-cell responses that will lead to the production of antibodies against the antigen. Graus et al. [85] suggested that the expression of HLA-DR in SGCs may facilitate an autoimmune reaction in the DRG in SSyn, but this idea was not followed up.

Direct evidence for a role for SGCs in SSyn is not available, but there is information on the presence of a specific autoantibody in SGCs of SSyn patients. Calponin is a calcium binding protein found in smooth muscle cells but also in other cell types. Birnbaum et al. [86] found that the level of calponin-3 autoantibody in the serum of SSyn patients was significantly higher than in controls. They also obtained immunohistochemical evidence for the presence of calponin 3 in SGCs (but not in neurons) in rat DRG. They suggested that, by binding to the cytoskeleton, calponin may play a regulatory role in the development of disorders such as osteoarthritis and seizures. Unfortunately, this preliminary work has not been pursued further. One can speculate that the binding of calponin autoantibodies to SGCs may alter their function, which, in turn, would influence neuronal activity. An obvious experiment will be to find out whether this binding can activate SGCs, thereby contributing to pain.

### 2.3. Fibromyalgia

Fibromyalgia (FM) is common pain disorder, affecting 2.7% in the general population, with a greater occurrence (4:1) in females than males [87]. FM patients suffer from chronic musculoskeletal pain, mechanical hypersensitivity, high sensitivity to cold, fatigue, and cognitive difficulties. FM is a controversial issue because of the lack of specific biological and instrumental biomarkers and comorbidity with several other disorders [88]. The diagnosis of FM is challenging, because the symptoms may resemble those of other pain conditions such as Raynaud and Sjögren syndromes [89]. The mechanism underlying FM is obscure, but it is believed that central sensitization is an important factor [90].

One approach to understand the pathophysiology of FM is that it is an autoimmune disease, as there have been indications that FM is highly prevalent in patients with other autoimmune diseases, such as RA [91]. Significant progress in testing this idea took place recently with the discovery that the injection of immunoglobulins (IgGs) from serum from FM patients (FM IgG) into mice lowered the withdrawal threshold for mechanical stimulation [92]. This pain-like behavior persisted for several days. Similar results were obtained for cold sensitivity. In these and in the following experiments, FM IgG was compared with IgG from normal humans. The authors found that FM IgG sensitized nociceptors in mice and concluded that there is a passive transfer of hypersensitivity from human to mice.

Using Western blot, Goebel et al. [92] found that FM IgG accumulated in the DRG of treated mice but not in the brain or spinal cord. Interestingly, they also found by immunohistochemistry that, in the treated mice, FM IgG was localized in SGCs of DRG but not in sensory neurons. Similarly, an in-vitro study showed that FM IgG labeled SGCs in human DRG. Spinal cord cells did not display FM IgG immunoreactivity. Furthermore, SGCs from FM IgG-treated mice showed increased immunostaining for of the glial activation marker GFAP. There was no evidence for neuronal death in DRG from treated mice. Also, FM IgG did not increase cytokine levels and did not produce inflammation in the FM IgG-treated mice.

To learn about the neurophysiological consequences of FM IgG injection, Goebel et al. [92] recorded action potentials from the saphenous nerve in skin-nerve preparations from mice. They found that the firing threshold in response to mechanical stimulation of the skin was lower in preparations from FM IgG-treated mice. Also, the proportion of cold sensitive nerves was greater in FM IgG mice.

In a further study, it was found that the binding of FM IgG to SGCs increased with the severity of the disease [93]. This suggests that a high level of anti-SGC IgG is an important factor in the generation of pain in FM. Moreover, the results indicate the importance of peripheral mechanisms in pain generation in FM. These results correlate well with the extensive research on pain models in rodents, which have indicated a role of SGCs in pain. Still, many details are missing for a full understanding of FM. For example, it is not known how IgGs bind to SGCs and how this binding activates these cells. Also, we do not know how SGC activation leads to neuronal sensitization. On the basis of animal studies, it was suggested that chemical signaling from SGCs by ATP and cytokines, combined with gap junctional communications, is a candidate mechanism that still needs to be tested in human ganglia [3].

Tracey [94] discussed some of the consequences of the work of Goebel et al. [92] and pointed out that it “provides insight into a mysterious disease affecting millions of people and offers a solid and promising insight into just how to begin developing effective therapeutic strategies for a severe, chronic, uncurable illness”. Among the possible therapeutic strategies that this work inspires are reducing FM IgG levels by antigen-specific adsorption after the relevant epitopes have been identified. Another approach is to inhibit FM IgG binding to SGCs or to inhibit their functional activation. If the mode of signaling between activated SGCs and neurons is clarified, interfering with this mechanism can also have a therapeutic potential.

In summary, although further validation of the conclusions above and extension of the experiments are required, it is quite possible that FM will cease to be a ‘mysterious’ disorder and will be classified as an autoimmune disease.

### 2.4. Paraneoplastic Neuropathies

Paraneoplastic neuropathies (PN) are a group of rare disorders that occur in cancer patients in which the immune system releases antibodies that attack normal cells in the nervous system in addition to attacking cancer cells [95,96]. This may lead to a variety of neurological disorders of the PNS and CNS. In Lambert–Eaton syndrome, cancer cells release antibodies that bind to Ca^2+^ channels in neuromuscular junctions, causing muscle weakness [97]. Paraneoplastic neuropathies may be associated with pain, as happens when cancer cells release an antibody against voltage-gated K^+^ channels (VGKC) complex, which may cause hyperexcitability of the sensory neurons [98,99]. Research in this field has focused on neurons, but SGCs also carry K^+^ channels, whose blockade can increase neuronal activity [100,101], and, therefore, it makes sense to examine whether SGCs are a target for VGKC-complex antibodies. There are a few reports on the possible involvement of SGCs in PN: In DRG of patients with paraneoplastic ganglioradiculoneuritis, there is death of neurons and proliferation of SGCs (nodules of Nageotte) [38]. In paraneoplastic encephalomyelitis (PEM), there is evidence that damage to DRG is an important factor in the generation of pain [98], but there are no details on the involvement of SGCs. In DRG obtained post-mortem from patients with paraneoplastic ganglionitis due to small cell lung carcinoma, there was an increased expression of MHC class II in SGCs [102], which may enable SGCs to mount an immune response against antigens.

Intercellular adhesion molecule-1 (ICAM-1) is a member of the immunoglobulin superfamily of adhesion molecules. Bernal et al. [103] found that SGCs in DRG displayed a greater level of ICAM-1 and -3 than controls. This included SGCs in nodules of Nageotte and that surround apparently normal neurons, which may indicate that the changes in SGCs precede those in the neurons and may play a role in the neuronal pathology. Augmented levels of ICAM were also observed in certain CNS regions. The authors suggested that the augmented ICAM could contribute to the damage to DRG in patients with PEM. In contrast, in a rat model of sciatic nerve injury, ICAM-1 was present mostly in neurons and to a small degree in SGCs. Clearly, there is a need for studies that will focus on the possible role of SGCs in pain in PN, and the first step should be to test whether SGCs are activated in DRG from PN patients and in animal models of this disorder.

It was suggested that TNF-α, as well as other cytokines released by immune cells that invade the DRG, contribute to neuropathic pain in PN models [98]. There is evidence from animal studies that TNF-α that is released from activated SGCs and endoneurial macrophages in the DRG might initiate and maintain neuropathic pain [104].

### 2.5. Neurodegenerative Diseases

Neurodegenerative diseases (e.g., Alzheimer’s, Parkinson’s, Huntington’s) are associated with chronic pain, but the role of sensory ganglia or SGCs have not been explored in these disorders. Some progress has been made in the study of other, less common diseases, as described below.

#### 2.5.1. Friedreich Ataxia

**Figure 6:**
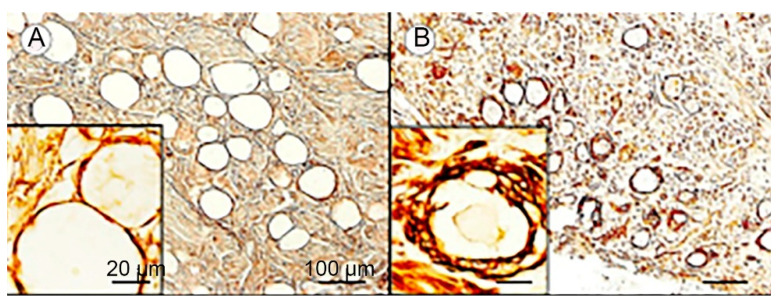
Satellite glial cells and neurons are altered in Friedreich ataxia (FA). (**A**) Section of DRG from a normal person labeled for the protein laminin. Note the thin SGC envelope around the neuron. (**B**) Section of DRG from an FA patient showing abnormal onion-like layers and projections outside the neuron–SGC unit. From Koeppen et al. [35]. Reproduced with permission from Reprinted under Creative Commons Attribution (CC-BY) license.

**Figure 7:**
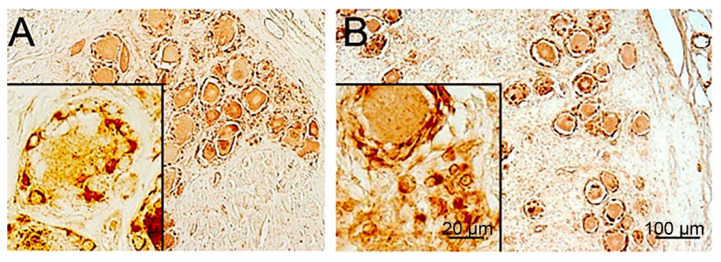
Immunostaining for glial fibrillary acidic protein (GFAP) of human DRG. (**A**) Normal control. (**B**) FA patients. Both images show positive staining for GFAP, but the total amount of the protein is considerably greater in the DRG from the FAS patient. For further details, see Koeppen et al. [35]. Courtesy of Prof. A. Koeppen, Veterans Affairs Medical Center, Albany, NY, USA.

Thanks to the efforts of Prof. Arnulf Koeppen and his coworkers, Friedreich ataxia (FA) is the human disease where SGCs have been the most thoroughly characterized. FA is a lethal genetic disease characterized by deficiency in the mitochondrial protein frataxin. Patients suffer from impaired movement and cardiac functions (the main cause of death), sensory loss, and skeletal abnormalities [105,106]. The main neuropathological feature in FA is a degeneration of neurons in the cerebellum and spinal cord. However, histological studies have shown abnormalities in the DRG as well. Koeppen et al. [35] carried out an immunohistochemical investigation of DRG cells from FA patients in comparison with healthy persons, and their observations can be summarized as follows: 1. There is a reduction in neuronal size (Figure 6). 2. SGCs in FA undergo an increase in number and size (hyperplasia and hypertrophy). They form disorganized onion-like layers encircling neurons, which are similar to the picture in VZV (Figure 3). This makes the SGC envelope around the neurons much thicker (Figure 6 and Figure 7). 3. In FA, SGCs that surround small neurons show a greater abundance of the gap junction protein connexin 43. In view of the numerous glial processes that develop in FA, coupling between SGCs in envelopes of different cells is more likely to occur, as found in mice after nerve injury or inflammation [3,107]. 4. In FA, SGCs showed a higher level of the mitochondrial marker ATP5B and of mitochondrial frataxin. 5. In both control and FA, SGCs contain several functionally important proteins: metabotropic glutamate receptors 2 and 3 (mGluR2/3), excitatory amino acid transporter 1 (EAAT1), glutamine synthetase, and rectifying potassium channel Kir4.1. This holds also for the structural proteins S100, laminin, and vimentin, and also for the activation marker GFAP (Figure 7). Examining the images, it appears that the intensity of immunostaining is similar between FA and controls. However, because of the much larger volume of the SGCs in FA, the level of these proteins is much greater. The functional significance of these observations is not clear. It was found that the volume of the SGC sheath increases with the neuronal volume [2], which was explained by the greater metabolic demands of larger cells, which require a larger number of SGCs. However, in FA, the neurons shrink, which makes it difficult to explain the greater SGC volume.

Augmentation of some of the proteins mentioned above has been observed in rodent models of injury and inflammation, and their possible contribution to chronic pain have been discussed [3] and will not be pursued here; still, the observations on the mitochondria deserve comment. Koeppen et al. [35] have suggested that the “mitochondrial abundance is a compensatory, and likely maladaptive, response to oxidative stress, energy deficiency, or both”. As mitochondria are a source of reactive oxygen species, the augmented number of these organelles in SGCs may cause further damage to DRG cells. This raises issues of how SGCs regulate the neuronal environment and whether they supply metabolites to the neurons. It was recently found that SGCs can regulate the amount of some neurotransmitters (ATP, Glutamate, GABA) that reach the neurons in mouse DRG and TG [108]. It is known that SGCs and neurons can interact through the release of cytokines, growth factors, and ATP [3,8], but it is still not clear whether mitochondrial changes can affect these interactions.

An important conclusion from the results of Koeppen et al. [35] is that normal SGC function fails in FA and that atrophy and inflammatory invasion of neurons are secondary events to this failure. This means that the disease process in FA affects SGCs independently and is not solely due to atrophy of DRG neurons. It is conceivable that frataxin deficiency affects SGCs directly, but this and many other questions about SGCs’ function and pathology still remain to be answered. The work of Koeppen et al. [35] can serve as a guide for investigating the possible changes in SGCs and for understanding disease mechanisms in humans.

SGC activation and several other molecular changes in them that occur in FA resemble the observations in rodent pain models. However, pain is not a prominent symptom in FA. This may be explained by the nature of the changes in neuronal properties that take place in FA. In the pain models, the initial event is neuronal insult, which induces SGC activation and other changes. In contrast, in FA, pathological changes in SGCs may drive neuronal dysfunction, as discussed above.

#### 2.5.2. Machado–Joseph Disease

Machado–Joseph Disease (MJD), also called spinocerebellar ataxia type 3, is a hereditary neurodegenerative disease characterized by severe clinical manifestations and premature death [109]. Patients suffer from motor problems but also from non-motor and extra-cerebellar symptoms. including sleep disorders, cognitive deficits, and pain [110]. Several CNS regions, mainly the cerebellum and substantia nigra, are affected in MJD, but neurons’ SGCs in DRG are also affected. Immunostaining for the glial marker S-100 showed the formation of abnormal multiple layers of SGCs around neurons and also in residual nodules (Nageotte nodules) [111]. The significance of this observation is still unclear.

#### 2.5.3. Amyotrophic Lateral Sclerosis (ALS)

ALS is the most common neurodegenerative disease affecting motor neurons (MNs). Although the selective cell death of MNs is a key feature of ALS, other tissues and organs may also contribute to the clinical manifestations of the disease, which include sensory neuropathy and pain [112,113]. ALS is characterized by mutations in the superoxide dismutase 1 gene (SOD1), and a useful animal model for ALS is the transgenic SOD1G93A mouse. Ruiz-Soto et al. [114] studied DRG in this model and found that SOD1G93 immunofluorescence was virtually absent in controls but was prominent in SOD1G93 mutant mice. The SOD1G93A level was low in the neurons in the mutant mice but high in SGCs. They also observed abnormal accumulation of lipid droplets and lysosome-related structures in SGCs, which reflect disrupted lysosomal homeostasis leading to lysosomal storage pathology in these cells. Ruiz-Soto et al. [114] suggested that SGCs in the mutant mice undergo an induction of oxidative stress, which in turn may lead to abnormal firing in sensory neurons that may contribute to the clinical manifestations of ALS, including pain. That work again supports the importance of cross-talk between SGCs and neurons, which may become abnormal under pathological conditions. These preclinical observations should lead to studies on ALS patients.

### 2.6. Diabetic Neuropathy

Diabetes mellitus (DM) has become a global epidemic; for example, the prevalence of DM type 2 is about 10% in China and India [115]. Neuropathy is a common complication in DM type 1 and 2, afflicting at least 50% of the patients and is difficult to treat [116]. A frequent consequence of diabetic neuropathy (DNP) is pain. Research on this topic has focused mostly on pathological changes in axons, but there is evidence that functional changes in sensory neurons can contribute to diabetic pain. In animal models of DM, sensory neurons show a misexpression of Na^+^ channels [117] as well as numerous molecular abnormalities [118], indicating that sensory ganglia are highly suitable targets for research and therapy of DNP. There is evidence that, in the streptozotocin model of DM 1 in rodents, SGCs are activated, as assayed by the upregulation of GFAP [119], and also undergo morphological changes [120]. Some biochemical changes in SGCs in rodent models of DM were also found [3,121,122]. In spite of the global nature of DM and the severity of its clinical consequences, there is no information on SGCs in DM patients. This can be a fertile field of study and may bring about progress in the understanding and treatment of diabetic pain.

### 2.7. Systemic Inflammation (Sickness Behavior)

Systemic inflammation is a common human disease. The symptoms associated with this disorder are called ‘sickness behavior’ and include depression and pain [123]. A major factor in sickness behavior is increased cytokine levels, and the underlying mechanisms are believed to be central. A simple way to induce sickness behavior is to inject LPS intraperitoneally, and this was performed in both human and animals. Animal studies based in this method showed that DRG were strongly affected, including the upregulation of pro-inflammatory cytokines and cyclooxygenase-2 in lumbar DRG, which may be involved in the behavioral changes [124]. Studies on SGCs have shown that a single LPS injection activated SGCs, augmented their responses to ATP, and increased SGC–SGC and neuron–neuron dye coupling by gap junctions [125]. Intraperitoneal injection of gap junction blockers raised the pain threshold back to the control level, suggesting a role for gap junctions in the pain behavior. Some of the effects persisted for at least 4 weeks [77]. LPS injection in humans causes inflammation and the familiar symptoms of sickness behavior [126], but changes in sensory ganglia have not been explored.

## 3. Conclusions

Ideally, studying human disease mechanisms is performed on human tissues. However, this approach is limited by ethical, technical, and logistical issues. As discussed above, a creative way to avoid some of these difficulties is to combine human and animal work. The experiments where Zerboni et al. [50] grafted human DRG into SCID mice to learn about VZV infection of human DRG is one example. Further examples are the studies where IgGs from patients with FM were injected into mice and showed that SGCs may be crucial in this disorder. In-vitro incubation of animal ganglia with serum from patients is another promising approach. These studies, as well as work on post-mortem material, indicate that SGCs may be major players in a variety of human disorders. The author hopes that this review will encourage further investigations of the disorders mentioned here, and many others, such as obesity, complex regional pain syndrome, alcoholism, and autoimmune diseases, such as lupus, inflammatory bowel diseases, and psoriasis.
